# Editorial: The bone/bone marrow microenvironment: A hub for immune regulation of the tumor cells fate

**DOI:** 10.3389/fimmu.2022.1019489

**Published:** 2022-08-31

**Authors:** Konstantin Horas, Ciro Menale, Antonio Maurizi

**Affiliations:** ^1^ Department of Orthopaedic Surgery, Koenig-Ludwig-Haus, University of Wuerzburg, Wuerzburg, Germany; ^2^ Department of Clinical Medicine and Surgery, University of Naples Federico II, Naples, Italy; ^3^ Department of Biotechnological and Applied Clinical Sciences, University of L’Aquila, L’Aquila, Italy

**Keywords:** bone cancer, bone microenvironment, immune regulation, bone metastases, bone marrow microenvironment

The bone/bone marrow microenvironment is a fascinating yet complex biological system. It contains both haematopoietic and mesenchymal cells of multiple lineages, a sinusoidal blood supply, the bone marrow stroma and the bone extracellular matrix (1). Under physiological conditions, resident bone cells (osteoblasts, osteoclasts and osteocytes) interact with other temporary present cell types such as myeloid and immune cells, platelets, bone marrow endothelial and haematopoietic cells and bone marrow-derived mesenchymal stem cells in a well-orchestrated manner (1). Furthermore, hormones, locally acting growth factors and cytokines engage with these cells to varying degrees, thus, impacting on the bone microenvironment (2, 3). Together, this complex interplay allows for a balanced bone metabolism within the bone microenvironment. Concomitantly, this fertile milieu provides an ideal soil that attracts cancer cells (4). Upon arrival in bone, cancer cells adapt to the new microenvironment in several ways (5, 6). Notably, some cancer cells start to grow and thrive within the bone microenvironment while others enter a long-lived dormant/quiescent state (7). As soon as cancer cells start to expand in bone they are known to parasite and control the local environment by exploiting resident osteoblast and osteoclasts to foster their own prosperity. Ultimately, the bone microenvironment may be considered an airport hub regulating the “tumour cell trafficking” redirecting them to either cellular dormancy/quiescence or to growth as bone metastases and potentially spreading into distal tissues. In this context, immune cells are believed to play a pivotal role impacting on the tumour cells fate.

This Research Topic focuses on the role of immune cells regulating the tumour cells behaviour in the bone microenvironment.

Li et al. highlight the role of the RANKL-RANK axis in the immune microenvironment and bone metastases and review data on the role of regulatory mechanism of immunity in bone metastases. In this review, multiple up-to-date studies have been discussed and the authors illustrate how the changes in the immune microenvironment contribute to bone metastases, providing a highly modular platform potentially applicable to a broad range of cancer bone metastases. Further, they explain how these findings have been validated and implicated into the clinical setting by e.g. the development of the humanised monoclonal antibody against RANKL (denosumab) that has been demonstrated to successfully prevent bone loss, skeletal-related-events and bone metastases in cancer patients . The bone marrow niche is a structure within the intra-trabecular spaces of spongious bones and of the cavity of long bones that houses adult haematopoietic stem cells (HSCs). Within the niche, HSCs interact with various cell types such as osteoblasts, endothelial cells, macrophages, and mesenchymal stromal cells (MSCs), which maintain HSCs in a quiescent state or sustain their proliferation, differentiation, and trafficking, depending on body needs. Novel findings have demonstrated that cancer cells impact on the bone marrow niche and are able to disrupt the bone microenvironment in such a way as to promote the initiation and progression of several malignancies. Granata et al. highlight recent advances regarding the BM niche composition and functionality in normal and under malignant conditions, as well as the therapeutic implications of the interplay between its diverse cellular components (mainly focusing on recent data of pro- and anti-tumorigenic MSC-functions) and malignant cells. Specifically, the authors underlined that disruption of the delicate BM microenvironment promotes the initiation and progression of myelodysplastic syndromes (MDS) and myeloproliferative neoplasms (MPN), also favouring resistance to pharmacological therapies. Marie and Bonnelye provide an update on how estrogens impact on bone immune cells and explain their consequences on bone homeostasis, metastasis settlement into bone and tumour progression. Moreover, they address the role of an orphan nuclear receptor ERRalpha (“Estrogen-receptor Related Receptor alpha”) on macrophages and T lymphocytes, and as an immunomodulator in bone metastases. Furthermore, there is increasing evidence that immune cells in the bone microenvironment affect the transformation and progression of multiple myeloma (MM). Isola et al. conducted a gene expression analysis of bone marrow cells (collected *via* bone marrow aspiration) from patients with smouldering multiple myeloma (SMM, pre-malignant condition) and compared them to samples of patients with monoclonal gammopathy of undetermined significance (MGUS) or symptomatic MM. They found an upregulation of genes encoding for key molecules in cytotoxicity in bone marrow cells of patients with SMM compared to both MGUS and symptomatic MM. As a conclusion, these changes in genes associated with exhausted cytotoxic T cells may be relevant as biomarkers to better characterise the progression risk of asymptomatic patients with SMM. Several studies suggest that cell-to-cell communication between myeloma and immune cells *via* tumour cell-derived extracellular vesicles (EV) play a key role in the pathogenesis of MM. For this reason, Lopes et al. analysed bone marrow immune alterations induced by MM-derived extracellular vesicles (EV). Their findings demonstrate that MM-derived EV promote immunosuppression by modulating the phenotype of lymphoid cells with an increase in the expression of the IC PD-1 and CTLA-4, accompanied by a decrease in CD27 expression, thus facilitating myeloma cell escape and progression.

Collectively, this Research Topic provides an overview and new insights into the role of immune cells in regulating tumour cell behaviour in the bone microenvironment. Although numerous recent studies have helped to better understand the complex network underlining the cellular interactions of cancer cells within the bone microenvironment, many open questions remain that will need to be addressed with future research.

## Author contributions

KH wrote the first draft of the manuscript. All authors contributed to the manuscript, read, and approved the submitted version

## Conflict of interest

The authors declare that the research was conducted in the absence of any commercial or financial relationships that could be construed as a potential conflict of interest.

## Publisher’s note

All claims expressed in this article are solely those of the authors and do not necessarily represent those of their affiliated organizations, or those of the publisher, the editors and the reviewers. Any product that may be evaluated in this article, or claim that may be made by its manufacturer, is not guaranteed or endorsed by the publisher.
